# Outcomes in patients with infections and augmented renal clearance: A multicenter retrospective study

**DOI:** 10.1371/journal.pone.0208742

**Published:** 2018-12-10

**Authors:** Yasumasa Kawano, Junichi Maruyama, Ryo Hokama, Megumi Koie, Ryotaro Nagashima, Kota Hoshino, Kentaro Muranishi, Maiko Nakashio, Takeshi Nishida, Hiroyasu Ishikura

**Affiliations:** 1 Department of Emergency and Critical Care Medicine, Fukuoka University Hospital, Fukuoka, Japan; 2 Department of Emergency and Critical Care Center, Kochi Health Sciences Center, Kochi, Japan; Azienda Ospedaliero Universitaria Careggi, ITALY

## Abstract

Recently, augmented renal clearance (ARC), which accelerates glomerular filtration of renally eliminated drugs thereby reducing the systemic exposure to these drugs, has started to receive attention. However, the clinical features associated with ARC are still not well understood, especially in the Japanese population. This study aimed to evaluate the clinical characteristics and outcomes of ARC patients with infections in Japanese intensive care unit (ICU) settings. We conducted a retrospective observational study from April 2013 to May 2017 at two tertiary level ICUs in Japan, which included 280 patients with infections (median age 74 years; interquartile range, 64–83 years). We evaluated the estimated glomerular filtration rate (eGFR) at ICU admission using the Japanese equation, and ARC was defined as eGFR >130 mL/min/1.73 m^2^. Multivariable logistic regression analysis was performed to identify the independent risk factors for ARC and to determine if it was a predictor of ICU mortality. In addition, a receiver operating curve (ROC) analysis was performed, and the area under the ROC (AUROC) was determined to examine the significant variables that predict ARC. In total, 19 patients (6.8%) manifested ARC. Multivariable logistic regression analysis identified younger age as an independent risk factor for ARC (odds ratio [OR], 0.94; 95% confidence interval [CI], 0.91–0.96). However, ARC was not found to be a predictor of ICU mortality (OR, 0.57; 95% CI, 0.11–2.92). In addition, the AUROC of age was 0.79 (95% CI, 0.68–0.91), and the optimal cut off age for ARC was ≤63 years (sensitivity, 68.4%; specificity, 78.9%). The incidence of ARC was, therefore, low among patients with infections in the Japanese ICUs. Although younger age was associated with the incidence of ARC, it was not an independent predictor of ICU mortality.

## Introduction

Infections remain a leading cause of mortality among intensive care unit (ICU) patients despite numerous clinical advances [1]. For patients with infections, one of the most important approaches is to start treatment with adequate doses of appropriate antibiotics early on [2]. Recently, a phenomenon of augmented renal clearance (ARC), which influences the renal elimination of antibiotics, is gaining recognition [3, 4]. ARC occurs in a hyperdynamic state, caused by inflammatory mediators in critical conditions, and refers to an enhanced renal elimination of circulating solutes [5]. Since ARC accelerates glomerular filtration of renally eliminated drugs, it leads to a reduced systemic exposure to these drugs [6–8]. Previous studies have shown the prevalence of ARC to be about 14–80% in ICU patients [9–17]. Since creatinine clearance (CrCl) is not routinely measured in the ICUs for daily treatments [4], there is the challenge to detect ARC simply by the estimated glomerular filtration rate (eGFR), which is calculated using various formulas (such as the Cockcroft–Gault [CG] equation [18], Modification of Diet in Renal Disease [MDRD] Study equation [19], and the Chronic Kidney Disease Epidemiology Collaboration [CKD-EPI] equation [20]) in clinical practice worldwide. In addition, a Japanese eGFR equation is used to calculate eGFR [21] in the Japanese ICU settings. However, only a few studies on ARC that evaluated eGFR by using the Japanese equation have been reported. Furthermore, evidence for relevant clinical outcomes in Japanese ICU patients with ARC is still limited. The aims of this study were to determine the clinical characteristics and outcomes of patients with infections who also had ARC evaluated on the basis of eGFR calculated using the Japanese equation in ICU settings.

## Materials and methods

### Setting

This retrospective, two-multicenter, observational study was performed at two tertiary level ICUs in Japan, from April 2013 to May 2017. This study was approved by the Institutional Ethics Committees of the Fukuoka University Hospital and Kochi Health Sciences Center (numbers 17-10-03 and 171085). The opportunity was made for opting-out, instead of giving informed consent individually. All data were fully anonymized for this study.

### Study population

The inclusion criteria for study admission were as follows: age ≥18 years, suspected infection and receiving antibiotics for therapeutic use. Patients were excluded if at the time of admission there was evidence of pregnancy, suspicion of rhabdomyolysis, serum creatine kinase (CK) concentration >5000 IU/L, renal impairment (serum creatinine [S_Cr_] >1.1 mg/dL), or a history of renal replacement therapy.

### Data collection and definition

The medical records collected at the time of admission were reviewed to investigate demographic and laboratory data, including age, sex, history of diabetic conditions, serum levels of albumin, CK, and creatinine, Acute Physiology and Chronic Health Evaluation (APACHE) II scores, Sequential Organ Failure Assessment (SOFA) scores, ventilation variables, source of infection, the initial empirical choice of antibiotics including combined antibiotic therapy for infections, results of the blood culture, number of ICU-free days determined on day 28, and ICU mortality.

ARC was defined as eGFR >130 mL/min/1.73 m^2^ [5]. An eGFR for diagnosing ARC in this study was calculated using a 3-variable Japanese equation [21].

For men: eGFR (mL/min/1.73 m^2^) = 194 × [S_Cr_ (mg/dL)]^−1.094^ × age^−0.287^

For women: eGFR (mL/min/1.73 m^2^) = 194 × [S_Cr_ (mg/dL)]^−1.094^ × age^−0.287^ × 0.739

Although, the eGFR calculated by CG, MDRD, and CKD-EPI was also evaluated in comparing the proportions of patients falling into various GFR ranges according to each equation.

The SCr levels were determined by laboratory analysis using an enzymatic method.

### Statistical analysis

Results are expressed as mean (± standard deviation [SD]) or median (interquartile range [IQR]) for continuous data, and as a percentage for categorical data. The Student t test or Mann–Whitney U test and chi-square test were used for continuous and categorical data, respectively. Multivariable logistic regression analysis was performed to identify the independent risk factors for ARC and to determine if ARC can predict ICU mortality. Because serum albumin levels and diabetic conditions have been shown to influence tubular creatinine secretion [22, 23], these factors were included as explanatory variables in a multivariable analysis for the risk factors of ARC. In addition, age and male sex, both of which are known risk factors for ARC, were also included as explanatory variables in this analysis [14, 15]. Furthermore, the explanatory variables in another multivariable analysis for the predictor of ICU mortality were determined from the ARC status and any variables with a p-value of less than 0.1 in the univariate analysis. The odds ratio (OR) and 95% confidence interval (CI) were calculated. Moreover, a receiver operating curve (ROC) analysis was performed, and the area under the ROC (AUROC), was determined to evaluate the accuracy of the significant variables in predicting ARC. All tests were two-tailed, and a p-value of <0.05 was considered statistically significant.

All statistical analyses were performed by using the EZR software program (Saitama Medical Center, Jichi Medical University, Saitama, Japan) [24], which is a graphical user interface for the R software program (The R Foundation for Statistical Computing, Vienna, Austria). More precisely, it is a modified version of R commander, which was designed to add statistical functions frequently used in biostatistics.

## Results

### Characteristics and clinical data

Demographic, laboratory, treatment, and outcome data for the enrolled patients are shown in Table 1.

**Table 1:** Baselines characteristics, laboratory, therapeutic, and outcome data.

Variables	All patients (n = 280)	ARC (n = 19)	Non-ARC (n = 261)	p value ^a^
Age (years), median (IQR)	74 (64–83)	46 (28–68)	75 (65–83)	<0.05
Sex, male, n (%)	145 (51.8)	9 (47.4)	136 (52.1)	0.81
Mechanical ventilation, n (%)	113 (40.4)	12 (63.2)	101 (38.7)	0.05
Diabetes mellitus, n (%)	47 (16.8)	4 (21.1)	43 (16.5)	0.54
APACHE Ⅱ score, median (IQR)	20 (16–25)	23 (19–27)	20 (16–24)	0.06
SOFA score, median (IQR)	5 (3–7)	6 (4–8)	5 (3–7)	0.17
Serum albumin (g/dL), mean (SD)	2.9 (0.76)	2.8 (0.97)	2.9 (0.75)	0.52
Serum CK (IU/L), median (IQR)	71.5 (35–155)	42 (25.5–76)	74 (35–159)	0.05
Serum creatinine (mg/dL), median (IQR)	0.7 (0.6–0.9)	0.3 (0.3–0.37)	0.74 (0.6–0.9)	<0.05
Positive blood culture, n (%)	71 (25.4)	3 (15.8)	68 (26.1)	0.42
Site of infection, n (%)				
Lung	117 (41.8)	13 (68.4)	104 (39.8)	<0.05
Abdomen	80 (28.6)	3 (15.8)	77 (29.5)	0.29
Skin and soft tissue	40 (14.3)	3 (15.8)	37 (14.2)	0.74
Urinary tract	17 (6.1)	-	17 (6.5)	-
Surgical site	7 (2.5)	-	7 (2.7)	-
Heart	5 (1.8)	-	5 (1.9)	-
Central nerve system	4 (1.4)	-	4 (1.5)	-
Catheter	2 (0.7)	-	2 (0.8)	-
Unknown	8 (2.9)	-	8 (3.1)	-
Antibiotic, n (%)				
Carbapenems	137 (48.9)	6 (31.6)	131 (50.2)	0.15
Piperacillin-tazobactam	63 (22.5)	6 (31.6)	57 (21.8)	0.39
Ampicillin-sulbactam	45 (16.1)	6 (31.6)	39 (14.9)	0.1
Linezolid	13 (4.6)	1 (5.3)	12 (4.6)	0.61
Glycopeptides	13 (4.6)	-	13 (5)	-
Clindamycin	9 (3.2)	2 (10.5)	7 (2.7)	0.12
Fluoroquinolones	7 (2.5)	-	7 (2.7)	-
Cephalosporins	6 (2.1)	1 (5.3)	5 (1.9)	0.35
Macrolides	6 (2.1)	-	6 (2.3)	-
Daptomycin	3 (1.1)	1 (5.3)	2 (0.8)	0.19
Others	8 (2.9)	-	8 (3.1)	-
ICU-free days on Day 28, median (IQR)	21 (12–25)	19 (12–23)	22 (12–25)	0.23
ICU mortality, n (%)	27 (9.6)	2 (10.5)	25 (9.6)	0.7

ARC, augmented renal clearance; IQR, interquartile range; APACHE, Acute Physiology, and Chronic Health Evaluation; SOFA, Sequential Organ Failure Assessment; SD, standard deviation; CK, creatine kinase; ICU, intensive care unit.

^a^ The p values were evaluated by comparison between patients with and without ARC.

We enrolled 280 patients in this study (median age, 74 years [IQR, 64–83 years], 51.8% men). The median APACHE II score was 20 (IQR, 16–25), and the median SOFA score was 5 (IQR, 3–7). Positive blood culture was reported for 71 (25.4%) of the patients. The most common site of infection was the lung (41.8%), and about half the patients received carbapenems (48.9%) for their treatment. While ICU mortality rate was 9.6%, ARC was seen in only 19 patients (6.8%). The age, S_Cr_ and incidence of lung infections were significantly different between patients with and without ARC (all p <0.05), though the ICU mortality rates among the two groups were not significantly different (p = 0.7).

The patients with positive blood culture, with and without ARC were selected and compared for clinical data (Table 2). In about half the cases, the detected pathogen was gram-positive coccus (39/71, 54.9%). Including ICU mortality, there were no variables that showed a significant difference between patients with and without ARC.

**Table 2:** Comparison of bacteriological and outcome data in patients with positive blood culture, with and without ARC.

Variables	ARC (n = 3)	Non-ARC (n = 68)	p-value
Microbiological examination, n (%)			
Gram positive coccus	2 (66.6)	37 (54.4)	1.0
Gram-negative rods	-	20 (29.4)	-
Gram-positive coccus and Fungus	1 (33.3)	-	-
Fungus	-	5 (7.4)	-
others	-	6 (8.8)	-
ICU-free days on Day 28, median (IQR)	19 (9.5–21)	23 (13.8–25)	0.24
ICU mortality, n (%)	1 (33.3)	5 (7.4)	0.24

ARC, augmented renal clearance; ICU, intensive care unit; IQR, interquartile range.

The proportions of patients falling into various eGFR ranges as assessed by the Japanese, CG, MDRD, and CKD-EPI equations are shown in Table 3. The number of patients with an eGFR >130 mL/min/1.73 m2 was found to be different according to each equation; 19 patients (6.8%) were identified using the Japanese equation, 28 patients (10%) using the CG equation, 57 patients (20.4%) with the MDRD equation, and 13 patients (4.6%) using the CKD-EPI equation.

**Table 3:** Proportions of patients falling into various eGFR ranges as assessed by the Japanese, CG, MDRD, and CKD-EPI equations.

	Japanese n (%)	CG^a^ n (%)	MDRD n (%)	CKD-EPI n (%)
eGFR >130 mL/min/1.73 m^2^	19 (6.8)	28 (10)	57 (20.4)	13 (4.6)
130≥ eGFR >90 mL/min/1.73 m^2^	54 (19.3)	55 (19.6)	96 (34.3)	109 (38.9)
90≥ eGFR >60 mL/min/1.73 m^2^	124 (44.3)	98 (35)	106 (37.9)	136 (48.6)
60≥ eGFR >30 mL/min/1.73 m^2^	83 (29.6)	97 (34.6)	21 (7.5)	22 (7.9)
30≥ eGFR >15 mL/min/1.73 m^2^	0	2 (0.7)	0	0
eGFR ≤15 mL/min/1.73 m^2^	0	0	0	0

eGFR, estimated glomerular filtration rate; CG, Cockcroft–Gault; MDRD, Modification of Diet in Renal Disease; CKD-EPI, Chronic Kidney Disease Epidemiology Collaboration.

^a^ The CG equation was calculated with body surface area correction.

### Risk factors and predictive values for ARC

Multivariable logistic regression analysis performed for four variables (age, male sex, history of diabetes mellitus, and serum albumin), indicated only younger age to be an independent risk factor for ARC (OR, 0.94; 95% CI, 0.91–0.96) (Table 4).

**Table 4:** Multivariable logistic regression analysis for risk factors of ARC.

Variables	OR (95% CI)	p-value
Age	0.94 (0.91–0.96)	<0.05
Male sex	0.82 (0.3–2.29)	0.71
Diabetes mellitus	1.95 (0.55–6.9)	0.3
Serum albumin	0.66 (0.35–1.26)	0.21

ARC, augmented renal clearance; OR, odds ratio; CI, confidence interval.

We performed the ROC analysis to evaluate age as a predictive factor for ARC. The AUROC of age was 0.79 (95% CI, 0.68–0.91), and the optimal cut off age for ARC was ≤63 years (sensitivity, 68.4%; specificity, 78.9%) (Table 5).

**Table 5:** Age as a predictor of ARC using the receiver operating curves.

	AUROC	95% CI	Optimal cut off values	Sensitivity (%)	Specificity (%)	PPV (%)	NPV (%)
Age (years)	0.79	0.68–0.91	63	68.4	78.9	76.4	71.4

ARC, augmented renal clearance; AUROC, area under the receiver operating curve; CI, confidence interval; PPV, positive predictive value; NPV, negative predictive value.

### Predictor of ICU mortality

The comparison of clinical data between survivors and non-survivors are shown in Table 6. The following variables were significantly different between survivors and non-survivors: mechanical ventilation, APACHE II scores, SOFA scores and serum albumin (all p <0.05).

**Table 6:** Comparison of clinical data between survivors and non-survivors.

Variables	Survivors (n = 253)	Non-survivors (n = 27)	p value
ARC status, n (%)	17 (6.7)	2 (7.4)	0.7
Age (years), median (IQR)	74 (64–83)	73 (65–79)	0.64
Sex, male, n (%)	119 (53)	16 (40.7)	0.31
Mechanical ventilation, n (%)	95 (37.5)	18 (66.7)	<0.05
Diabetes mellitus, n (%)	39 (15.4)	8 (29.6)	0.1
APACHE II scores, median (IQR)	20 (16–24)	24 (17–28.5)	<0.05
SOFA scores, median (IQR)	5 (3–7)	7 (5–8)	<0.05
Serum albumin (g/dL), mean (SD)	2.9 (0.75)	2.6 (0.85)	<0.05
Serum CK (IU/L), median (IQR)	72 (35–155)	71 (42–141)	0.84
Serum creatinine (mg/dL), median (IQR)	0.7 (0.6–0.9)	0.72 (0.5–0.9)	0.58
Positive blood culture, n (%)	65 (25.7)	6 (22.2)	0.82
Site of infection, n (%)			
Lung	101 (39.9)	16 (59.3)	0.06
Abdomen	75 (29.6)	5 (18.5)	0.27
Skin and soft tissue	37 (14.6)	3 (11.1)	0.78
Urinary tract	17 (6.7)	-	-
Surgical site	5 (2)	2 (7.4)	0.14
Heart	4 (1.6)	1 (3.7)	0.4
Central nerve system	4 (1.6)	-	-
Catheter	2 (0.8)	-	-
Unknown	8 (3.2)	-	-
Antibiotic, n (%)			
Carbapenems	124 (49)	13 (48.1)	1.0
Piperacillin-tazobactam	55 (21.7)	8 (29.6)	0.34
Ampicillin-sulbactam	43 (17)	2 (7.4)	0.27
Linezolid	13 (5.1)	-	-
Glycopeptides	11 (4.3)	2 (7.4)	0.36
Clindamycin	7 (2.8)	2 (7.4)	0.21
Fluoroquinolones	6 (2.4)	1 (3.7)	0.51
Cephalosporins	5 (2)	1 (3.7)	0.46
Macrolides	6 (2.4)	-	1.0
Daptomycin	3 (1.2)	-	1.0
Others	8 (3.2)	-	1.0

ARC, augmented renal clearance; IQR, interquartile range; APACHE, Acute Physiology, and Chronic Health Evaluation; SOFA, Sequential Organ Failure Assessment; SD, standard deviation; CK, creatine kinase.

Multivariable logistic regression analysis was performed for six variables including ARC status, mechanical ventilation, APACHE II scores, SOFA scores, serum albumin and lung infection. No variables, including ARC status (OR, 0.45; 95% CI, 0.08–2.46), were found to be an independent predictor of ICU mortality (Table 7).

**Table 7:** Multivariable logistic regression analysis for a predictor of ICU mortality.

Variables	OR (95% CI)	p-value
ARC status	0.45 (0.08–2.46)	0.36
Mechanical ventilation	2.36 (0.97–5.75)	0.06
APACHE Ⅱ scores	1.05 (0.99–1.12)	0.1
SOFA scores	1.05 (0.9–1.23)	0.52
Serum albumin	0.62 (0.34–1.1)	0.11
Lung infection	1.85 (0.76–4.52)	0.18

ICU, intensive care unit; OR, odds ratio; CI, confidence interval; ARC, augmented renal clearance; APACHE, Acute Physiology and Chronic Health Evaluation; SOFA, Sequential Organ Failure Assessment.

## Discussion

This study demonstrates a very low incidence of ARC in patients with infections and no renal impairment on the first hospital day. Our results show that younger age is an independent risk factor for ARC. In addition, the optimal cut off age for identifying ARC patients was ≤63 years. However, there was no significant difference in the ICU mortality rates between patients with and without ARC, even in those with a positive blood culture. Additionally, ARC status was not an independent predictor of ICU mortality.

The percentage of patients with ARC in this study was *6*.*8*%, which is much lower than the reported rates in previous studies [9–17]. There could be three reasons for this difference. First, different CrCl cutoff values have been used for diagnosing ARC in the previous studies. Because many previous studies have defined ARC as patients with a CrCl >130 mL/min/1.73 m^2^, we defined ARC as patients with an eGFR >130 mL/min/1.73 m^2^ in this study [4]. However, while several previous reports have diagnosed ARC in cases with CrCl >130 mL/min/1.73 m^2^ [5, 9–11, 14–17], other studies have set the cutoff for CrCl at >120 mL/min/m^2^ [12, 13] and >160 mL/min/1.73 m^2^ [8]. Though the best definition of ARC in the critically ill is still unknown, these different CrCl cutoff values could possibly account for the varying ARC prevalence rates reported by different studies including ours. Second, the different populations could account for the varying results among different studies. The risk factors for ARC have been reported to be young age, male sex, trauma and lower illness severity [14, 15]. The incidence of ARC reported, therefore, depends on how many subjects in a study have one or more of these risk factors. For instance, our study involved many elderly patients, with a median age of 74 years and the oldest patient was 106 years old. The relatively fewer number of young patients could, therefore, account for the lower prevalence of ARC seen in our study. Third, we assessed ARC retrospectively without a measurement for urinary CrCl. Instead, we used the eGFR values which were calculated by a Japanese equation for evaluating ARC. This Japanese equation has been reported to underestimate the GFR in ICU settings [10]. In addition, other commonly used formulas (such as CG, MDRD, and CKD-EPI) for eGFR worldwide have also been shown to underestimate the actual measured CrCl in ARC patients [16, 17]. As shown in our study, the different assessment techniques used, such as using various equations for diagnosing ARC, might have yielded different results for the incidence of ARC.

A multivariable logistic regression analysis showed that younger age was an independent risk factor for ARC, and a ROC analysis showed that the AUROC of age and cut off age were 0.79 and ≤63 years for screening ARC patients, respectively. Interestingly, these results are consistent with those of a previous study which evaluated ARC in patients by measuring CrCl for 8 h in Japanese ICU settings [10]. However, age cannot help identify the ARC patients accurately. Age should be used only as a screening tool for identifying ARC patients, and it is necessary to evaluate GFR for diagnosing ARC.

We found that ARC was not associated with ICU mortality. Although many studies have shown that patients with infections and ARC have enhanced renal elimination of renally cleared antibiotics and therefore a reduced exposure to these drugs [6–8], there are still no studies showing the relationship between ARC and mortality [12, 25, 26]. The only adverse outcome, shown for patients with ARC in the previous studies, was the therapeutic failure of the antibiotics used [27–29]. If sepsis patients turn decline in status during their clinical course, a multi-organ failure including acute kidney injury (AKI) cannot be avoided [2]. Previous studies have demonstrated that AKI on admission was associated with both ICU and hospital mortality in sepsis patients [30]. However, ARC has been shown to occur in patients who had a lower illness severity without AKI [15]. Since these populations, who were at risk of ARC, also tended to have low mortality from the beginning, it might have been difficult to show the correlation between ARC status on ICU admission and mortality. Nevertheless, therapeutic failure in patients with ARC might be an important outcome that physicians should pay attention to because it might be associated with the eventual acquisition of resistance to antimicrobial agents [28]. In addition, ARC might be associated with the prophylactic failure of antibiotic therapies given to trauma, burn, postoperative and immunocompromised patients.

This study showed that no variables were independent predictors of ICU mortality. Interestingly, the severity of illness, evaluated by the SOFA and APACHE II scores, was not associated with ICU mortality. Although the reason for this lack of association is not clear, the underlying disease and comorbidities, which are not evaluated enough by these scoring systems, could have an effect on ICU mortality in a population that was not seriously ill and had a median SOFA score of 5 points in this study.

This study has a number of limitations. First, this was a retrospective study, although it included two multicenters. Second, renal function was not evaluated by measuring the urinary or plasma clearance of an ideal filtration marker such as inulin [31]. Third, this study aimed to evaluate the ARC status on ICU admission only. Although the frequency of cases with ARC is high during the first day of ICU stay, it has been reported even during the first 7 days of ICU stay [12]. Fourth, there was no evaluation of whether the empirical antimicrobial treatments, their doses and period used were appropriate for the patients with infections in this study. Finally, in this study, the S_Cr_ levels were evaluated by an enzymatic method, which was different from the Jaffe method used in a previous study. S_Cr_ levels measured by the Jaffe method have been shown to be higher than those by the enzymatic method [32]. Since the creatinine levels were used to diagnose ARC, the difference in the methods used for their estimation could have potentially impacted the findings of our study.

## Conclusion

This study found that the incidence of ARC was low in Japanese ICU patients with infections and normal S_Cr_ levels on the day of admission. Younger age was found to be the only independent risk factor for ARC. Although age might be a useful screening tool for estimating ARC in patients, ARC itself was not a predictor of ICU mortality. Further studies are needed to determine the association between ARC and the adverse clinical outcomes, especially therapeutic failure/ prophylactic failure, in ICU settings.
